# Analysis of Feeder Bus Network Design and Scheduling Problems

**DOI:** 10.1155/2014/408473

**Published:** 2014-01-02

**Authors:** Mohammad Hadi Almasi, Sina Mirzapour Mounes, Suhana Koting, Mohamed Rehan Karim

**Affiliations:** Center for Transportation Research, Civil Engineering Department, Faculty of Engineering, University of Malaya, 50603 Kuala Lumpur, Malaysia

## Abstract

A growing concern for public transit is its inability to shift passenger's mode from private to public transport. In order to overcome this problem, a more developed feeder bus network and matched schedules will play important roles. The present paper aims to review some of the studies performed on Feeder Bus Network Design and Scheduling Problem (FNDSP) based on three distinctive parts of the FNDSP setup, namely, problem description, problem characteristics, and solution approaches. The problems consist of different subproblems including data preparation, feeder bus network design, route generation, and feeder bus scheduling. Subsequently, descriptive analysis and classification of previous works are presented to highlight the main characteristics and solution methods. Finally, some of the issues and trends for future research are identified. This paper is targeted at dealing with the FNDSP to exhibit strategic and tactical goals and also contributes to the unification of the field which might be a useful complement to the few existing reviews.

## 1. Introduction 

Many people usually use public transportation systems to reach their destination; however, others employ personal vehicles. If the transport system is unable to attract travellers, private transport usage will be increased. Nowadays, to prevent the increasing rate of private transports entering to city centres, effective alternatives of travel modes need to be offered [1]. In addition, a good performance in public transport has been recognized among the potential means to reduce air pollution, decrease energy consumption, and improve mobility and traffic congestion.

General public transportation planning process can be categorized into five steps as the following: (1) network design, (2) frequency setting, (3) timetabling, (4) vehicle scheduling, and (5) crew scheduling [2]. In the recent publications, there has been an increasing interest in the first three steps and also basic aspects of the planning process, which are known as strategic (step 1) and tactical (steps 2 and 3) planning process.

The intermodal system has been a challenging and controversial issue in the field of public transportation. In order to improve complicated public transportation system, intermodal system can be beneficial. This system usually consists of the integrated rail line and a number of feeder routes connecting to the transfer stations. The rail line providing an effective and convenient mode can carry the large number of travellers, while the feeder bus routes provide a means to transport passengers from bus stops to train stations. This paper will focus on performing a review of research works for a specific problem arising in a feeder network design, with the goal of providing readers with a broader and more complete insight on the subject. The aim of this study is to evaluate and analyze intermodal system problems in public transportation. The main concern is how to increase efficiency of feeder route design and coordinated schedules so as to minimize cost. An improved integrated intermodal system might lead to a decrease in total cost and an increase in profits via keeping shorter routes and removing duplicated routes. Furthermore, higher quality services for passengers can be provided. The structure of this paper is as follows: presenting the problem description for each of its subproblems and characteristics. It will then go on to create the classification of existing problems based on approaches and the solution methods. In addition, evaluation and analysis are presented to describe the characteristics and limitations for reviewing other works. For a broader understanding of the domain, different classifications are offered to show recent policy and developments affecting feeder network problems. Finally, the concluding remarks are offered to present problems and opportunities for future works.

## 2. Problem Description

Passengers gathered at bus stops located in the service area wish to access their destination. They travel by feeder bus to any rail stations and then proceed to the city centre or their destination [1]. This procedure occurs almost all over the world several times a day, and it includes so many challenges and issues. This study effort has been made to go further into details of these problems. In this segment, four subproblems of FNDSP will be presented and it will introduce the studies done on these subproblems.

### 2.1. Data Preparation

Data preparation includes the area's topology, origin-destination (OD) matrices, fleet size, and more information such as bus and train operating costs, route length, speed, demand, and so forth.

The road network, bus stops, stations, and transfer zones define the area's topology [3]. And also the travel times, distance between rail stations, and bus stops or demand can be specified by OD matrices. Sometimes, geographic information system (GIS) and various shortest path algorithms are utilized for calculating the travel time and distances of OD matrices. Kim and Jeong [4] compared the performance of several shortest path algorithms and developed an approximation approach to OD matrices generation.

Vehicle data contains types of available buses which can have different capacities. Bus fleet describes the vehicle size. The available fleet size and bus capacities are very important to determine the service frequencies. Finally, detailed information is determined according to the problem of the study area, objectives, and constraints.

### 2.2. Feeder Bus Network Design Problem (FNDP)

Feeder bus network design is the first and most important step in bus transport planning procedure. The network design problem consists of determining a set of bus routes in a specific area, through the given travel demand, the area's topology characteristics, and set of objectives and constraints [3].

The route structure design is becoming an important input to the subsequent decision making processes and will affect later planning steps, significantly, which is explained in the following section.

#### 2.2.1. Feeder Route Generation

Feeder routes link residential complexes to railway stations [5]. A good design of route network can increase the efficiency of the feeder bus system and decrease the total cost of supplying the transit service. The users would like to have a bus network with more coverage area and high accessibility in the service area. Their perspective of a good service area is a feeder network with more direct through trips and highly demand satisfying. On the other hand, the operation's costs should be reduced by keeping the total route length within a certain bound. Thus the main challenge of the route network design is to be able to give a good and efficient alternative at a reasonable computation time. The feeder route network design problem can be solved by building initial solution using the contraction algorithm, followed by improving the existing solutions by means of applying a local search algorithm.

One of the construction heuristics for building initial solutions is a sequential building method, proposed by Kuah and Perl [6]. This method is adopted from sequential saving approach for Multi-Depot Vehicle Routing Problem (MDVRP). In another study Martins and Pato [1] expanded the research by Kuah and Perl [6] and created the initial solution by applying the sequential savings. Their research suggested a two-phase building method to generate the initial solution. Shrivastav and Dhingra [7] proposed Heuristic Feeder Route Generation Algorithm (HFRGA). This algorithm was greatly guided by the demand matrix developed by Hadi Baaj and Mahmassani [8]. Metaheuristic methods are also applied for initial population. Genetic algorithm (GA) for an initial population at random was used by Chien et al. [9]. Nevertheless, a random selection of nodes might not be a good selection for generating initial routes. Therefore, Kuan et al. [10, 11] employed the concept of delimiter, proposed by Breedam [12].

Pradhan and Mahinthakumar [13] on their paper described parallel implementations that includes performance analyses of two prominent graph algorithms (i.e., Floyd-Warshall and Dijkstra) used for finding the all-pairs shortest path for a large-scale transportation network. Their article also includes the derivation of the computational time for the different parallel implementations of these two graph algorithms.

The technique used in the feeder bus route generation is indicated in Table 1. In Table 1, “M” stands for “mathematical,” “H” for “heuristic,” and “Me” for “metaheuristic.”

After building initial solutions, improvements can be implemented on the routes. There are a lot of optimization methods to improve the solutions.

### 2.3. Feeder Bus Network Scheduling Problem (FNSP)

The problems of feeder bus scheduling can be categorized into three type groups, consisting of:

- timetabling, including departure times from all stops and stations served by the routes in the network,
- frequency setting, determining the feeder bus frequency for every route in the network,
- timetable and frequencies, applying timetable and frequency setting simultaneously for each route, with regard to the set of objectives and constraints.

**Figure 1:**
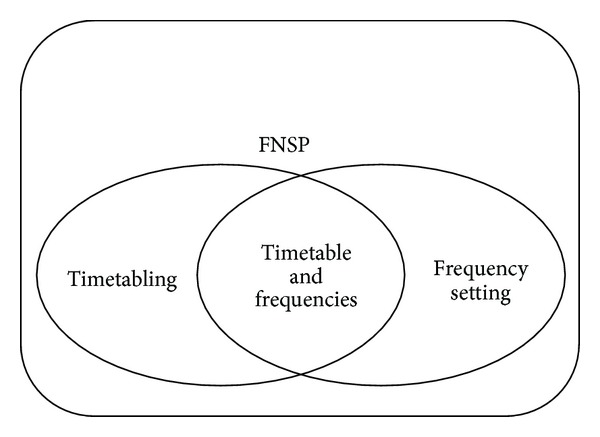
The schematic categorization of FNSP.

The schematic illustration of the three categories of FNSP is presented in Figure 1.

A suitable scheduling design can supply a sufficient standard service to satisfy the users. In addition, suitable scheduling contributes to reduction in fleet size and operators' cost, subsequently leading suppliers to be satisfied [3]. From the literature review it is evident that frequency setting problems have been mostly used for development scheduling problems.

## 3. Problem Characteristics

In this section, it has been tried to categorize FNDSP based on various characteristics. There are several ways to achieve these aims, but we mainly focus on the realistic aspects of the problem.

Table 2 shows the classified criteria of problem characteristics, and the important points of these criteria are described in the following subsections. In Table 3 the summary of criteria pertaining to some of the literature is presented.

### 3.1. Demand Pattern

In terms of passenger demand, it is usually assumed to be fixed or inelastic, for simplicity. Fixed demand may be reasonable for systems at which passengers are insensitive or independent of service quality or price. However, elastic demand can probably be variable, due to sharing or competition of the public transport. And rising rate of mobility demand will be significant factor to the efficiency of urban transportation modelling [14].

For feeder bus, two types of travel demand patterns, namely, many-to-one and many-to-many, are measured. The many-to-one demand pattern is discussed in several papers such as [6, 10, 11, 15–19], and so forth. This model refers to passengers traveling from multiple origins to a single destination. This is usually more applicable to feeder bus services which carry passengers to a common destination (central business district or a transfer station), and peak hour trips to and from CBD can be considered in this pattern. In most bus services, many-to-many demand pattern is considered whenever passengers have different origins and destinations. Kuah and Perl [6]; Chien and Schonfeld [15] considered many-to-one pattern model to find an optimal feeder bus network in the hope that it will include one bus stop for more than a route, each of which, serves demand for various sets of destination.

### 3.2. Objectives and Decision Variables

Many concepts can be considered for determining objectives, decision variables, and constraints, such as environmental, economic, political, and social factors. Transit agency policies for specifying stated factors are based on the importance of these factors. Cohen-Blankshtain and Feitelson [20] in a survey research showed that these criteria can be a number of land use policies and several transport measures. Therefore, it has been tried to discuss the objectives and list of decision variables in FNDSP in this section.

Most of the main objective in the literature is optimizing the problem to achieve minimum user and operator costs. Cost estimates can be required at early projects stages, before completion of a detailed design for several purposes including budgeting and feasibility decisions. This estimation is usually determined by parametric modelling technique [21].

Xiong et al. [19] developed a solution for the optimal routing design problem with the objective of minimizing the total cost, including user and supplier costs, considering passenger traffic demand and budget constraints. The optimization variables include route and headway.

Kuah and Perl [6] optimized routing structures and operating frequency to minimize the total bus operating costs. User costs include the bus riding time, waiting time, and rail costs. Golub et al. [22] showed in their research that bus improvements (special feeder concessions, new route, service configurations, etc.) and upgrading the train system are important objects to achieve a reduction in travel time for most OD pairs and overall safety improvements.

Transfer coordination is also a major goal in many studies. The global network schedule should take into account each transfer area and its associated routes in order to allow efficient transfer between lines in distance and time. Transferring between lines can be supported according to various criteria such as the number of travelers. Wirasinghe et al. [23] designed a coordinated rail/bus transit system that served peak travel between a metropolitan region and CBD. They obtained values of three related variable (interstation spacing, feeder bus zone boundary, train headways) so as to minimize the total operator and user cost. Area coverage is one of the important objectives to measure the percentage of the estimated demand which can be served by public transport. This rate can be calculated in different ways and however generally is dependent on characteristics including route length, bus stop, density, and route spacing [24–26]. A maximum distance from the stopping place of the public transport was chosen by the following technical rule, for example, “communication systems of towns, small towns, and villages” [27]. In a considerable number of studies walking distance of travelers is considered as a measure, the range of which is from 400 to 800 meters in both Euclidean or network distance [28].

The general objective of operators is to minimize the overall route length in view of a reduction in the number of vehicles and crew resources required to maintain a global transport system. And the number of lines alternatively can be considered. In addition, routes should not be too short or too long for profit reasons. In Table 2, the decision variables are considered as a special set of characteristics. Based on the review, in the most feeder network design studies there is an intention to consider bus route location (BRL) and bus frequency (BF) as decision variable.

### 3.3. Constraints

Different constraints have been considered for FNDSP as presented in Table 3. The following constraints were outlined by Kuah and Perl [6]:

 Route capacity (RC): maximum passenger on the route
 Maximum fleet size (N)
 Maximum route length (RL)
 Route feasibility (RF): meaning to determine the feasibility of the bus routes, which includes the following several subconstraints:
  - each bus node should be placed in a single route (many-to-one pattern);
  - the feeder-bus network may include a bus stop in more than a single route (many-to-many patterns);
  - each bus route must be linked to just one railway station;
  - each bus is assumed to halt at all the stops in its route;
  - each feeder bus route should be linked to one railway station;
  - bus stop can be assigned to a railway station only if a route which terminates at that station, passes through that bus stop.

Martins and Pato [1] also used these constraints as well as frequency bound (F).

Shrivastava and O'mahony [29] presented a hybrid approach for developing their model and found more efficient feeder routes by considering load factor, fleet size, and unsatisfied demand as constraints. In terms of fleet size, the vehicle schedule is created by a line run and transit network timetable, in which fleet size is a useful constraint to optimize resource usage in FNDSP. Demand constraint is also a critical issue. The demand can be considered unsatisfied when users' origin or destination is too far from the bus stops, or when direct feeder services are not sufficient. In general, if a trip requires more than two transfers, it is assumed that the user will switch to another mode of transport.

For some reason, transportation agencies might prefer to develop a network with a specific shape such as radial, rectangular, grid, and triangular shape [30].

In some studies, several of these constraints are considered as objective functions. For instance, Martins and Pato [1]; Mohaymany and Gholami [31] applied some limitations on frequency variable, considered as a constraint and simultaneously as an objective to minimize the total cost. Chowdhury and Chien [32] included the bus headway (BH) and train headway (RH) in their objective function, by applying limits on headways to achieve optimal range of headways in certain lines and areas of interest.

In general the candidate bus line should also satisfy other constraints such as the presence of overlapping bus lines and maximum allowable bus line directness [33]. Table 3 represents more detailed data.

## 4. Classification Based on the Approach 

Generally, previous approaches of FNDSP can be divided into two major groups: analytic and network approaches. These approaches differ in their purpose and have different advantages and disadvantages. They should be viewed as complementary rather than alternative approaches [34].

### 4.1. Analytical

Analytic models were developed to derive optimum relations between different components of the feeder bus network process. This approach starts by formulating the design objective as a continuous function with a set of design variables. It is assumed that the design variables are continuous, and the optimal values are obtained by using the optimal conditions according to objective function. The typical design variables are feeder route location, rail station spacing, and service frequencies.

**Figure 2:**
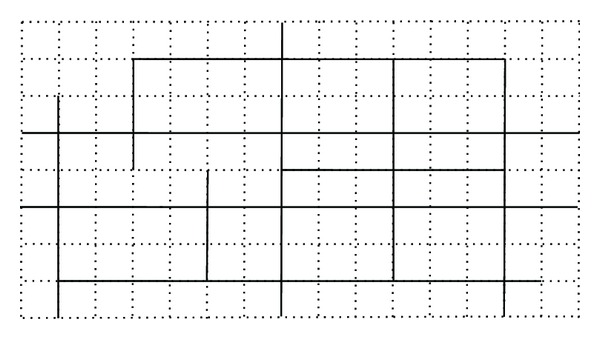
Example of an actual road network in an analytic model.

An analytic model needs a prespecified shape of the road geometry and a well-designed demand function presenting the distribution of demand in the service area. Numerous studies have attempted to explain analytic models, such as Wirasinghe [9, 15–17, 27, 30, 32, 35]. More studies are presented in Table 4. An example of the road configuration of an analytic model is shown in Figure 2.

This approach can process only small size or regularly shaped networks. So, number of the possible solutions increases substantially with increase in number of roads in network. If road network is simple, the usage in the model will only be limited to theoretical applications and may not be applied to real-world situations [18].

### 4.2. Network

Network approaches do not need prespecified shape of road geometry in the area. As a result, it is not limited to a simple network structure, but it can be applied to more complicated networks. Nodes and links signify the service area, and a route is represented by a sequence of nodes. Links present the segments of transport routes and usually travel times or distances are determined by links. Demand is assumed to be targeted at nodes [18]. Kuah and Perl [6], as first developers of network approach, resolved the FNDSP by means of mathematical programming models.

The network approaches adopted by Kuah and Perl [6], Martins and Pato [1], Shrivastav and Dhingra [7], Kuan et al. [10, 11], Mohaymany and Gholami [31], and so forth. More studies are shown in Table 4. In Table 4, “A” stands for “analytic” and “N” for “network”.

**Figure 3:**
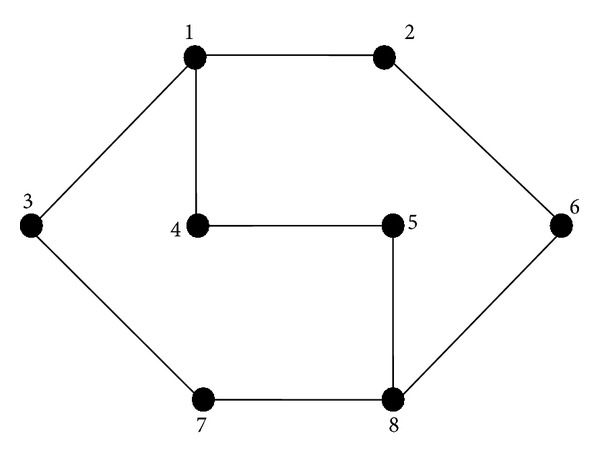
Example of a simple network model with eight nodes and nine links.

Past studies allocated discrete variables for demand and design element in network approach, leading to its capability to deal with larger problem sizes and more realistic situations. Furthermore, it can be divided into three groups: headway models, route structure models, combined headway, and route structure models. A simple example of the network approach is shown in Figure 3.

## 5. Classification Based on Solution Methods

The solution methods of problems can be categorized into four groups, namely, mathematical, heuristic, metaheuristic, and hybrid method. In this section we will explain these methods and analyze performance of each of them.

### 5.1. Mathematical Programming Methods

Wirasinghe [27, 35] used a mathematical method for designing a coordinated rail/bus transit system that served travel in peak hours between a metropolitan region and its CBD. The rail lines were assumed to be radial. Wirasinghe [35] optimized the zone boundary at which feeder buses should serve a rail line in order to minimize the sum of users and operating costs. It was applied to a given set of rail station spacing and constant train headways. Wirasinghe et al. [23] obtained values of three interrelated parameters, called station spacing, feeder bus zone boundary, and train headways in order to minimize the total cost.

In another study, Wirasinghe [36] applied mathematical method for analyzing a case of feeder bus access to a rail line on a rectangular street network. Feeder buses along parallel routes fed passengers to rail lines. In this study, rail station density, bus frequency, and route density were determined and led to minimization of total operator and user costs. As major assumption in this research, all passengers should walk to the nearest bus route. Moreover, Hurdle and Wirasinghe [37] extended the study of Wirasinghe [36] to include several feeder modes such as auto, bus, and bicycle. A simple algebraic formulation that shows the relationship between the station spacing and the various cost and demand parameters was developed. However, only the rail station spacing was optimized in this research.

Kuah and Perl [17] presented an analytic model for designing an optimal feeder bus network for accessing to an existing rail line. In order to minimize total cost, they applied mathematical method and avoided to combine stop spacing, route spacing and operating headway variables simultaneously, (see Table 4).

### 5.2. Heuristic Methods

From the literature review it is evident that heuristic approaches have been very popular for solving problems. These methods typically are examined when a specific problem is created by a mathematical formula. Usually, these methods contain linear and integer programming. Because of the flexibility of heuristic methods, a lot of documents published in recent years utilized them [38].

Heuristic methods have been used by Kuah and Perl [6] to quickly search for approximately good solutions by means of different sets of rules to construct routes in step-by-step and iterative procedures. Such methods were also used to local search improving techniques, such as displacement heuristics and exchange heuristics suggested by Perl [39].

Shrivastav and Dhingra [7] developed a heuristic algorithm for accessibility of feeder routes to suburban railway stations. This heuristic algorithm was the first part of a model which was developed to integrate the suburban stations and bus services. The second part was determined for optimizing coordinated schedules of the feeder bus services by using existing schedules of suburban trains. The proposed algorithm (HFRGA) was able to develop feeder routes to satisfy demands in various nodes. In addition, Chowdhury and Chien [32] proposed a model seeking for better coordination of the intermodal transit system. They applied a numerical search algorithm (Powell's algorithm) to solve the problem.

Chien [40] suggested specific feeder bus service to provide a shuttle service between a recreation center and a major public transportation facility. They proposed an integrated methodology (analytical and numerical techniques) for development and optimization of the decision variables.

It should be noted that in some of the studies heuristic methods have been used for initial building, in feeder bus network design problem, such as Kuah and Perl [6]; Kuan et al. [10, 11]; Martins and Pato [1]. More studies are presented in Table 1.

### 5.3. Metaheuristic Methods

In recent years, there has been an increasing amount of research on applying metaheuristic method to resolve the problems, such as simulated annealing (SA), GA, ACO, and so forth. These methods derive their concept from mathematics and physics, combined with biological evolution and artificial intelligence [1, 9].

Tabu search (TS) is one of the well-known metaheuristic methods, which is adopted by many researches. Martins and Pato [1] extended the work of Kuah and Perl [6] to improve previously proposed solutions. The initial solution was improved by using some heuristic procedures and generated a set of problems with real life situations. As a result, the simplest short-term version of TS provided better solutions and it can be one of the important heuristic methods in the future. In addition Kuan et al. [10] published a paper in which SA and TS are applied for resolving the problem. They generated several random tests to evaluate and compare the performance of their methods in terms of efficiency and accuracy of solutions. Thus, TS was more successful than SA in solving problem.

Chien and Yang [16] proposed an Exhaustive Search algorithm (ES) to optimize feeder bus route location and its operating headway in a given network. Moreover, Chien et al. [9] extended Chien and Yang's [16] study by presenting a GA to solve the problem. The GA started with an initial population and followed by improving route. The results of this study indicate that the optimum solutions discovered by ES and GA are identical. However, the computational time for a GA was significantly less than ES, especially for large or complicated networks.

In another study, Kuan et al. [11] described two metaheuristics methods (GA and ACO), for solving FNDSP. They have developed better algorithms to be able to provide a good solution for FNDSP in a reasonable period of calculation time. Moreover, in another study by Shrivastava and O'mahony [41], it was demonstrated that optimal feeder routes and schedules simultaneously by using GA in suburban service. The developed routes and schedules were optimized, but the whole demand was not satisfied since some of the nodes did not have good connectivity with other nodes in the study area.

Nikolic and Teodorovic [42] developed another metaheuristic algorithm to solve the transit network design problem. They applied Bee Colony Optimization (BCO) algorithm and tried to maximize the number of satisfied passengers, to minimize the total number of transfers, and to minimize the total travel time of all served passengers.

Mohaymany and Gholami [31] suggested an approach to solve multimodal feeder network design problem (MFNDP) with the objective of minimizing the total operator, user, and society costs. They used ACO for constructing routes and modifying the optimization procedure to identify the best mode and routes in the service area, (see Table 4).

### 5.4. Hybrid Methods

Hybrid method is another type of solution methods to solve FNDSP problems which combines the ability of different computational tools to resolve complicated problems. The research by Shrivastava and O'mahony [29] is one of the studies adopting hybrid method by using heuristic method to generate the potential routing and GA optimizing schedules of suburban trains. They offered a technique with two sub-models: routing for feeder buses, and schedule coordination.

Shrivastava and O'mahony [43] applied GA for route scheduling and network designing by repairing algorithm for satisfying design problem by offering a specialized heuristic algorithm. In another research, Shrivastava and O'mahony [44] presented a hybrid algorithm (SOHFRGA) which was an idea for developing public bus routes and coordinate schedules in suburban area. In the proposed research, GA and heuristic approach were combined to find optimized feeder routes with higher efficiency compared to those developed by other researchers using other approaches, (see Table 4).

## 6. Conclusions and Perspectives for Future Research

The objective of this review paper was to present descriptive analysis and classification of past research dealing with Feeder Bus Network Design and Scheduling Problem (FNDSP). Various aspects of reviewed studies have been grouped firstly by problem description, then problem characteristics, and finally solution approach and method.

As shown in Table 4, different studies have focused on various aspects of the problem and tried to define different objective functions, constraints, decision variables, and make different assumptions to simplify the problem. Hence, making critical comparison will be a difficult task. However, such diversity can be understandable, due to the fact that public transportation systems in different countries have different sets of rules and objectives for travelers' satisfaction. Also, passengers have different commuting behaviors and demand types, depending on their lifestyle and area zones of their residential area.

Since feeder bus network problem is an active research field, new policies by operators create new requirements and new challenges for planners and research groups. So, despite the effort made for FNDSP, the field still has a strong potential for future research. One of the most important fields to study could be cooperation and coordination between different levels of public transit such as train and feeders. Well-defined information and advanced schedule in intermodal system will lead to a high level of passengers' satisfaction.

Further study on travel demand is also needed. As discussed in demand approach section, most studies are restricted to many-to-one pattern and only few previous works considered many-to-many pattern. A many-to-one model refers to passengers traveling from multiple origins to a single destination. However, passengers may have different origins and destinations.

Modeling approaches should also be considered in future research. The improved approaches can increase the performance of the solutions by using other methods. Further modeling with multiple objectives and multiple criteria would be another area for further research. Since transit system is a multimodal, multiproblem, and multispectral activity, it involves different parts and activities such as policy making, planning, designing, infrastructure construction, and development. So, multiobjective models are important to be considered.

In addition, solution method should be considered to solve mathematical formulation. Although in recent years many heuristics and metaheuristics were proposed, and they were successfully implemented to FNDP, only a few studies on FNSP were performed. The development of metaheuristic methods has also made it possible to tackle large size problems more efficiently and to get high quality solutions for real life large size problems in a reasonable time. Hybrid methods also hold a lot of promises in terms of tackling problems that used to be intractable. Progress in solution methods is obviously a gate to the integration of all the previously mentioned interesting paths.

Finally, the literature survey also reveals that there has not been substantial work done on multimode feeder system providing service for an existing rail network. Most of the existing studies have been focused on the design of a single-mode network. Bovy and Hoogendoorn-Lanser [45] presented other findings on influence of multimodal trip attributes. In their study it was also focused on performance of different feeder mode, railway station type, and train service type. In the same way, further research can be focused on development of new approaches for multimode feeder network system.

## Figures and Tables

**Table 1:** Feeder route generation methods in the literature.

References	Initial building methods	Specify
Kuah and Perl [6]	H	Sequential savings
Martins and Pato [1]	H	Sequential savings and two-phase
Shrivastav and Dhingra [7]	H	HFRGA, Dijkstra's algorithm
Chien et al. [9]	Me	GA
Kuan et al. [10]	H	Delimiter algorithm, Breedam [12]
Kuan et al. [11]	H	Delimiter algorithm, Breedam [12]
Shrivastava and O'mahony [41]	H	*K*-path algorithm, Eppstein [46]
Shrivastava and O'mahony [43]	H	*K*-path algorithm, Eppstein [46]
Shrivastava and O'mahony [44]	H	*K*-path algorithm, Eppstein [46]
Shrivastava and O'mahony [29]	H	Dijkstra's algorithm
Mohaymany and Gholami [31]	Me	ACO
Gholami and Mohaymany [47]	Me	ACO

**Table 2:** Classification criteria from the problem perspective.

Criteria	Consideration
Demand pattern	Many-to-one Many-to-many

Problem scope	Morning Afternoon Peak

Decision variable	Feeder zone boundary (Z) Stop spacing (BS) Station spacing (RS) Bus frequency (BF) Bus headways (BH) Train headway (RH) Bus route density (BRD) Rail station density (RSD) Bus route location (BRL) Bus route length (BL) Rail line length (RL) Fleet size (N) Travel time (T) Mode (M)

Constraint	Load factor (LF) Geographic (G) Budget (BU) Demand bound (D) Bus route length (BL) Route feasibility (RF) Frequency bound (F) Bus headways (BH) Train headway (RH) Rail line length (RL) Maximum of fleet (N) Vehicle capacity (C) Travel distance (TD)

**Table 3:** Classification of the literature based on problem perspective.

References	Objective	Decision variable	Constraint	Demand pattern	Urban or suburban	Application area and scope
Wirasinghe [35]	Coordinate transit system (rail and bus)	Z	RH	Many-to-one	Urban	
Wirasinghe et al. [23]	Coordinate transit system (rail and bus)	Z, RS, RH		Many-to-one	Urban	Example, morning
Wirasinghe [36]	Coordinate operations	BRD, RSD, BF		Many-to-one	Urban	Calgary, peak
Hurdle and Wirasinghe [37]	Optimize rail station spacing	RS		Many-to-one	Urban	Calgary, peak
Kuah and Perl [17]	Optimal design for feeder bus	BS, BRL, BH		Many-to-one		
Kuah and Perl [6]	Optimal design for feeder bus	BRL, BF	BL, N, RF	Many-to-one Many-to-many	Suburban	Example benchmark, morning
Martins and Pato [1]	Optimal design for feeder bus	BRL, BF	BL, N, F, RF	Many-to-one	Suburban	Example benchmark, morning
Chien and Schonfeld [15]	Optimal design of integrated rail and bus	RL, BH, BS, RS, BRL		Many-to-many		Example
Chien and Yang [16]	Optimize feeder route location and headway	BRL, BH	G, BU, RC	Many-to-one	Suburban	Example
Shrivastav and Dhingra [7]	Development of routing and coordinated schedules	T	D, RL	Many-to-one	Suburban	Mumbai
Chien et al. [9]	Total welfare (operator and user cost)	BRL, BH	G, BU, RC	Many-to-one	Suburban	Example
Chowdhury and Chien [32]	Coordinated design of an intermodal transit system	BH, RH, BT	C, BH, RH	Many-to-one	Urban	Numerical example
Kuan [18]	Optimal design for feeder bus	BRL, BF	RL, N, RF	Many-to-one	Suburban	Example benchmark, morning
Kuan et al. [10]	Optimal design for feeder bus	BRL, BF	BL, N, RF	Many-to-one	Suburban	Example benchmark, morning
Chien [40]	Total welfare (operator and user cost)	BH, N, BRL	C, N, BU		Urban	Sandy Hook, park
Kuan et al. [11]	Optimal design for feeder bus	BRL, BF	BL, N, RF	Many-to-one	Suburban	Example benchmark, morning
Shrivastava and O'mahony [41]	Development of routing and coordinated schedules	BRL, BF	N, D, LF	Many-to-one	Suburban	Dublin
Shrivastava and O'mahony [43]	Development of routing and coordinated schedules	BRL, BF	N, D, LF	Many-to-one	Suburban	Dublin
Shrivastava and O'mahony [29]	Development of routing and coordinated schedules	BRL, BF	N, D, LF	Many-to-one	Suburban	Dublin, morning
Shrivastava and O'mahony [44]	Development of routing and coordinated schedules	BRL, BF	N, D, LF	Many-to-one	Suburban	Dublin, morning
Mohaymany and Gholami [31]	Optimize multimode feeder	BRL, BF, M	BL, N, F, BH, RS	Many-to-one	Suburban	Example benchmark, morning
Gholami and Mohaymany [47]	Optimize multimode feeder	BRL, BF, M	BL, N, F, BH, RS	Many-to-one	Urban	North of Tehran
Sivakumaran et al. [26]	Coordination of vehicle schedules in a transit system	BH, RH, BL		Many-to-one	Urban	An idealized network
Hu et al. [48]	Model for layout region of feeder	T	TD		Urban	Guangzhou
Ciaffi et al. [49]	Develop routing and scheduling simultaneously	BRL, BF	BL		Urban	Winnipeg and Rome, morning
Cipriani et al. [50]	Develop an operative tool in the bus system	BRL, BF	C, BL, F	Many-to-many	Urban	City of Rome
Xiong et al. [19]	Optimal routing problem	BRL, BH	D, BU	Many-to-one	Urban	Example

**Table 4:** Approaches and solution methods in the literature.

References	Approach model	Solution method	Specify
Wirasinghe [35]	A	M	
Wirasinghe et al. [23]	A	M	
Wirasinghe [36]	A	M	
Hurdle and Wirasinghe [37]	A	M	
Kuah and Perl [17]	A	M	
Kuah and Perl [6]	N	H	Displacement, exchange
Martins and Pato [1]	N	H	Displacement, exchange, TS
Chien and Schonfeld [15]	A		
Chien and Yang [16]	A	Me	ES
Shrivastav and Dhingra [7]	N	H	HFRGA
Chien et al. [9]	A	Me	ES, GA
Chowdhury and Chien [32]	A	H	
Kuan [18]	N	Me	TS, SA, GA, ACO
Kuan et al. [10]	N	Me	TS, SA
Chien [40]	A	H	
Kuan et al. [11]	N	Me	GA, ACO
Shrivastava and O'mahony [41]	N	Me	GA
Jerby and Ceder [51]	A	H	
Shrivastava and O'mahony [43]	N	Hy	GA and H
Shrivastava and O'mahony [44]	N	Hy	GA and H, SOHFRGA
Shrivastava and O'mahony [29]	N	Hy	GA and H
Mohaymany and Gholami [31]	N	Me	ACO
Gholami and Mohaymany [47]	N	Me	ACO
Sivakumaran et al. [26]	A	M	
Martinez and Eiro [52]	N	H	
Hu et al. [48]	A	M	
Ciaffi et al. [49]	N	Hy	GA and H
Cipriani et al. [50]	N	Hy	GA and H

“M” stands for “mathematical,” “H” for “heuristic,” “Me” for “metaheuristic,” and “Hy” for “hybrid.”
